# Association Between Recollections of *Shokuiku* During Elementary School, Current Well-Balanced Diets, and Health Behaviours in Japanese High School Students: A Sex-Stratified Study

**DOI:** 10.3390/nu18132108

**Published:** 2026-06-28

**Authors:** Etsuko Kibayashi, Makiko Nakade

**Affiliations:** 1 Department of Food and Nutrition Management, Sonoda University, Amagasaki 661-8520, Hyogo, Japan; 2 Department of Food Science and Nutrition, University of Hyogo, Himeji 670-0092, Hyogo, Japan; 3 Research Institute for Food and Nutritional Sciences, Himeji 670-0092, Hyogo, Japan

**Keywords:** *shokuiku*, nutrition education, well-balanced diet, high school student, lifestyle behaviour, sex difference

## Abstract

**Background/Objectives**: School-aged children in Japan receive food and nutrition education (*shokuiku*) to promote well-balanced dietary habits. However, among high school students, the association between current well-balanced diets and *shokuiku* during elementary school years has not been analysed. Herein, we examined the associations between recollections of *shokuiku* during childhood, current well-balanced dietary habits, and eating and lifestyle behaviours among Japanese high school students, with a particular focus on sex differences. **Methods**: Overall, 254 second-year high school students (56.3% female) at a public high school in the Hyogo Prefecture, Japan, were included. A hypothetical model was constructed using factors potentially associated with well-balanced dietary habits (i.e., consumption of balanced meals at least twice daily), including recollections of *shokuiku* during elementary school (education on nutritional balance based on the ‘three food groups’, major nutrients’ role, and breakfast importance) and current eating and lifestyle behaviours. Simultaneous sex-based multi-population analysis was performed. **Results**: The model demonstrated good fit (GFI = 0.944, AGFI = 0.903, CFI = 0.982, RMSEA = 0.036, and AIC = 145.174). Among female students, current well-balanced dietary habits showed significant positive associations with *shokuiku* (standardised estimate, female: 0.22, *p* = 0.005 vs. male: −0.07, *p* = 0.46), frequency of rice consumption (0.22, *p* = 0.016 vs. 0.13, *p* = 0.15), and eating meals with family (0.22, *p* = 0.003 vs. 0.36, *p* < 0.001). Conversely, bedtime (−0.28, *p* < 0.001 vs. −0.03, *p* = 0.72) showed a significant negative association. Among male students, only eating meals with family showed a significant positive association with current well-balanced dietary habits. **Conclusions**: Current well-balanced dietary habits among female high school students may be positively associated with *shokuiku*. Eating rice and meals with family was conducive to well-balanced dietary habits, unlike late bedtime.

## 1. Introduction

Well-balanced dietary habits, characterised by meals consisting of staple foods and main and side dishes, are associated with higher intakes of energy, protein, vitamins, and minerals and are more likely to meet the Japanese Dietary Reference Intakes [1]. The ‘Health Japan 21 (third term)’ [2] initiative, launched in April 2024, aims to increase the proportion of individuals who consume well-balanced diets at least twice daily on most days to 50% by 2032. However, according to a 2021 survey [3], only 36.4% of the population currently meets this recommendation, with low adherence among adults. These findings highlight the need to increase the prevalence of well-balanced dietary habits.

In Japan, the Basic Act on Food and Nutrition Education (*Shokuiku*) [4] was enacted and implemented in 2005. In the same year, the professionalisation of nutrition teachers began to provide food-related instruction in elementary and junior high schools. Consequently, an assessment of past *Shokuiku* efforts in educational settings is now required [5]. We previously reported that, among female Japanese students enrolled in a registered dietitian course, well-balanced dietary habits were positively associated with recollections of *shokuiku* during elementary school years [6]. However, no studies have examined the association among high school students of current well-balanced diets and *shokuiku* during elementary school years. Furthermore, as ‘Health Japan 21 (third term)’ [2] identifies health promotion based on a life-course approach as one of its four fundamental directions, it is crucial to clarify the potential influence of childhood *shokuiku* on high school students.

Regarding well-balanced dietary habits among high school students, factors beyond *shokuiku* may influence their current eating and lifestyle behaviours. For instance, among 20- and 30-year-old respondents in the 2016 Hyogo Dietary Survey, breakfast and rice consumption were associated with a higher frequency of well-balanced dietary habits [7]. Regarding eating meals with family, a survey of schoolchildren and university students reported that individuals who consumed less fast food and ate breakfast and dinner with their families also ate well-balanced diets more often at breakfast, lunch, and dinner [8]. Conversely, several lifestyle factors may negatively affect dietary quality. A study conducted in the United States reported that healthy adolescents aged 14–17 years with shorter sleep duration consume more carbohydrates, foods with high glycaemic load, sugary foods, and sweetened beverages, while consuming less vegetables and fruits [9]. Similarly, diet quality was reported to be low among Korean adolescents aged 14–17 years who frequently ate late at night [10]. However, no studies have comprehensively examined eating and lifestyle behaviours in adolescents or their associations with well-balanced dietary habits.

Therefore, we clarified the structural relationship of recollections of *shokuiku* during elementary school years, current eating and lifestyle behaviours, and well-balanced dietary habits among high school students. To the best of our knowledge, these relationships have not been comprehensively examined from a sex-specific perspective in Japan. The results are intended to inform future *shokuiku* initiatives for the next generation from a life-course perspective.

## 2. Materials and Methods

### 2.1. Study Design and Participants

This study included 278 second-year students enrolled in a public high school in a city in the Hyogo Prefecture in November 2021. The school was selected because its established community partnerships facilitated cooperation with the study. Of the 278 eligible students, 266 completed the survey questionnaire (response rate, 95.7%). After excluding questionnaires with missing data, 254 students (143 females and 111 males) were included in the final analysis (valid response rate, 95.5%). Second-year students were selected since they represented the middle year of the 3-year high school programme. First-year students were excluded because they had only recently entered high school and might not have fully adapted to the school environment. Conversely, third-year students were excluded because they were occupied with preparations for employment or higher education.

The survey was conducted on 17 November 2021, using a self-administered questionnaire designed to obtain cross-sectional responses on *shokuiku*-related conversations during elementary school years (nutritional balance of the ‘three food groups’, major nutrients’ role, and breakfast importance), current well-balanced dietary habits, and dietary and lifestyle behaviours. Students received written instructions on how to complete the questionnaire at distribution and were asked to independently answer the questions. The survey was anonymously conducted, and each student was assigned an identification number.

Before the survey, the students were provided with written information explaining that participation in the survey was voluntary and outlining the content of the study, including matters related to privacy. The students were asked for their understanding and cooperation in this study. Although the participants were 16–17-year-olds, parental/guardian consent on the consent statement was waived by the ethics committee. Therefore, their consent to participate in the survey was considered to have been obtained upon submission of the completed questionnaire. The Ethics Committee of the Sonoda University approved this study.

### 2.2. Measures

Participant characteristics were self-reported using questionnaire items on sex, height, weight, and physical activity level based on intensity (low, normal, and high) [11]. Support for healthy eating habits from family and others was assessed using the question ‘Does your family or others around you provide support for you to lead a healthier eating habit?’ with four response options: ‘Fairly much,’ ‘A little,’ ‘Almost none,’ and ‘Not at all.’ Participants were aged 16–17 years. Body size was assessed using weight-for-height, calculated as follows: actual weight (kg) − standard weight by height (kg)/standard weight by height (kg) × 100. Standard weight-by-height was determined according to the School Health Survey Report by the Ministry of Education, Culture, Sports, Science, and Technology [12].

Regarding well-balanced dietary habits, respondents were asked to answer the question ‘How many days a week do you eat a meal consisting of a staple, main dish, and side dishes at least twice a day?’ with four response options (Table 1).

The scale assessing recollections of *shokuiku* during elementary school years indicated their awareness, attitude, and practice in daily life regarding the contents of food and nutrition learned during elementary school, according to the degree of conversational experience based on their memories. The variables that comprised recollections of *shokuiku* during elementary school years were derived from a new textbook for fifth- and sixth-grade elementary school students approved by the Ministry of Education, Culture, Sports, Science, and Technology of Japan for elementary school home economics (Tokyo Shoseki) [13]. The three topics assessed were nutritional balance of the ‘three food groups’ (staples, such as cereal grains and potatoes; proteins, such as meat, fish, eggs, soybeans/soybean products, and dairy products; and side dishes, such as fruits and vegetables), the role of major nutrients, and the importance of breakfast. The students were asked to respond to the question ‘Did you have conversations about the following three items in your daily life when you were in elementary school?’ with four response options for each of the three items (Table 1).

Current eating and lifestyle behaviours included regular breakfast consumption, frequency of eating meals with family, snacking frequency (excluding late-night snacking), frequency of eating dinner within 2 h of bedtime, frequency of late-night snacking (after dinner and before bedtime), bedtime, and frequency of regular exercise (at least 30 min; Table 1). Bedtime was reported in hours and minutes and subsequently categorised into five groups. For all items presented in Table 1, scores were assigned in ascending order, with 1 and 4–5 points corresponding to the lowest and highest frequency, respectively. Regarding the frequency of rice consumption, respondents were asked to indicate the number of times per week they consumed rice at breakfast, lunch, and dinner.

### 2.3. Statistical Analysis

For comparisons of BMI, physical activity level, and support for healthy eating habits from family and others between the two sex groups, the chi-squared test was used for categorical variables, and Fisher’s exact test was used for expected frequencies of less than five. Although well-balanced dietary habits, recollections of *shokuiku* during elementary school years (three items), and current eating and lifestyle behaviours were measured using ordinal scales, these variables were treated as interval-scale variables, using Welch’s *t*-test. The approach was adopted because the study employed covariance structure analysis, which assumes interval-level data. To ensure consistency in data handling, the same treatment of these variables was applied throughout. Additionally, a sensitivity analysis using the Mann–Whitney U test was conducted to confirm that the choice of analytic approach did not affect the results.

**Figure 1:**
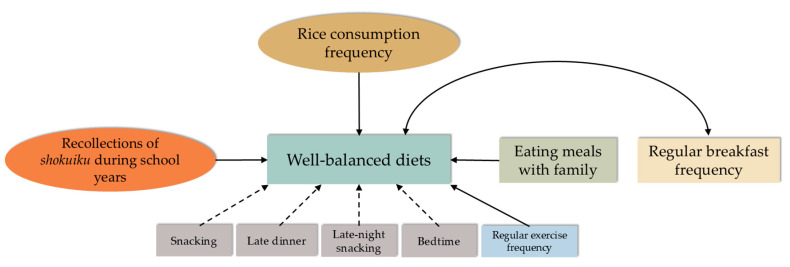
Initial hypothetical model of factors associated with well-balanced dietary habits. Bidirectional curved, solid, and dashed arrows indicate associations and positive and negative paths, respectively.

A hypothetical model was constructed using factors potentially associated with well-balanced dietary habits (consumption of at least twice daily), including recollections of *shokuiku* during elementary school years (past conversations about three items: nutritional balance of the ‘three food groups,’ major nutrients’ role, and breakfast importance) and current eating and lifestyle behaviours as potential facilitating or limiting factors (Figure 1). This model helps to verify the structural associations between recollections of *shokuiku* during elementary school years and current dietary and lifestyle habits related to well-balanced diet consumption. Facilitating factors included frequency of rice consumption, regular breakfast consumption, and regular exercise. Conversely, limiting factors included frequency of snacking, late dinners, late-night snacking, and later bedtimes. As an exploratory analysis, the hypothetical model was initially evaluated using covariance structure analysis as part of an overall structural examination of men and women. Subsequently, a simultaneous sex-based multi-population analysis was performed to assess model fit within each group and examine the invariance of components (i.e., whether the model structure was the same across groups) [14]. The model was iteratively refined by removing non-significant paths in the standardised estimates until an optimal fit was achieved based on path directions, standardised estimates, and fit indices, including the goodness of fit index (GFI), adjusted GFI (AGFI), comparative fit index (CFI), root mean square error of approximation (RMSEA), and Akaike’s information criterion (AIC). Model fit was considered acceptable when GFI, AGFI, and CFI indices were ≥0.9, RMSEA was ≤0.05, and AIC was relatively lower than that of other multiple models. Sample size was calculated using the null hypothesis RMSEA (ε0 ≤ 0.05) and alternative hypothesis RMSEA (ε1 = 0.1), with power of the close fit test of 0.8, model degrees of freedom of 52, and a significance level of 5%. With model degrees of freedom at 52, the calculated minimum sample size was 94 [15]. A critical ratio for differences between parameters of |Z| ≥ 1.96 was considered significant at the 5% level. Accordingly, statistical significance was set at *p* < 0.05. All statistical analyses were performed using IBM SPSS Statistics for Windows, version 29.0 (IBM Corp., Armonk, NY, USA).

## 3. Results

Table 2 lists participant characteristics and sex-based comparisons. Across the three categories of physical activity, a higher proportion of female students was classified as having a moderate activity level than that of male students (56.6% vs. 33.3%, respectively). However, a lower proportion of females was classified as having a high level of active exercise habits (20.3% vs. 54.1%)—indicating a significant difference between sexes (*p* < 0.001). No significant sex differences were observed in weight-for-height or in support for healthy eating habits from family and others. In contrast, a significant sex-based difference was observed in the frequency of consuming well-balanced meals at least twice daily (*p* = 0.008). A lower percentage of females reported consuming well-balanced meals 6–7 d per week compared with that of males (27.3% vs. 38.7%, respectively), while a higher percentage of females reported doing so ≤1 d per week (11.9% vs. 3.6%, respectively).

Table 3 lists the results of sex comparisons for each factor associated with well-balanced diets—recollections of *shokuiku* during elementary school years and current eating and lifestyle behaviours, including limiting factors. In the recollections of *shokuiku* during elementary school years group, no significant sex differences were observed in any of the three items: nutritional balance of the ‘three food groups,’ the role of major nutrients, and the importance of breakfast. Regarding current eating and lifestyle behaviours, females reported a lower mean weekly frequency of rice consumption at dinner (5.7 times) than that of males (6.5 times; *p* = 0.016). In addition, the proportion of females (21.7%) who regularly exercised for at least 30 min 6–7 d per week was lower than that of males (61.3%), indicating a significant difference between sexes (*p* < 0.001).

**Figure 2:**
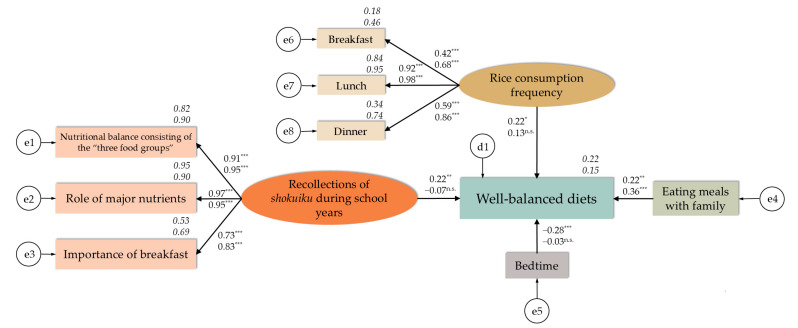
Associations between recollections of *shokuiku* during elementary school years, current well-balanced diets, and current eating and lifestyle behaviours based on sex (*n* = 254). Roman numerals in the path diagram indicate standardised estimates (shown next to straight arrows). Italicised numbers represent R^2^ values (coefficients of determination). Statistical significance was set at * *p* < 0.05, ** *p* < 0.01, and *** *p* < 0.001 (n.s., not significant). For each paired value, the upper and lower numbers correspond to females (*n* = 143) and males (*n* = 111), respectively. Simultaneous multi-populational analysis by sex indicated that the hypothetical model had acceptable goodness of fit (χ^2^ = 69.174, *df* = 52 [*p* = 0.056], GFI = 0.944, AGFI = 0.903, CFI = 0.982, RMSEA = 0.036, AIC = 145.174).

A multi-group structural analysis was conducted to examine whether the associations with well-balanced diets differed between sexes. In Figure 1, the results did not show acceptable goodness-of-fit indices (GFI = 0.863, AGFI = 0.811, CFI = 0.869, RMSEA = 0.061, and AIC = 408.253). Therefore, five current eating and lifestyle behaviour variables—regular breakfast consumption, snacking frequency, late dinner frequency, late-night snacking frequency, and regular exercise frequency—were removed from the initial model because they showed no significant associations with well-balanced dietary habits in either sex. Figure 2 shows the results of a simultaneous multi-group analysis, with a good model fit of GFI = 0.944, AGFI = 0.903, CFI = 0.982, RMSEA = 0.036, and AIC = 145.173. Among females, recollections of *shokuiku* during elementary school years (females: standardised estimate 0.22, *p* = 0.005, 95% confidence intervals 0.051–0.38 vs. males: −0.07, *p* = 0.46, −0.24–0.11) had a significant positive association with well-balanced dietary habits. Additionally, weekly rice consumption (0.22, *p* = 0.016, 0.020–0.41 vs. 0.13, *p* = 0.15, −0.059–0.29) and eating meals with family (0.22, *p* = 0.003, 0.076–0.36 vs. 0.36, *p* < 0.001, 0.19–0.52) as facilitating factors showed significant positive associations with well-balanced dietary habits. Bedtime (−0.28, *p* < 0.001, −0.44−0.11 vs. −0.03, *p* = 0.72, −0.24–0.20) showed a significant negative association with well-balanced dietary habits among females. In contrast, among males, eating meals with family was the only factor significantly positively associated with well-balanced dietary habits (0.36, *p* < 0.001, 0.19–0.52). Significant sex differences were observed in sex-based path coefficients, including recollections of *shokuiku* during elementary school years and bedtime to well-balanced dietary habits (critical ratios: 2.612 and −2.314 for females and males, respectively).

## 4. Discussion

This study developed a hypothesis model to investigate the structural associations between recollections of *shokuiku* during elementary school years and current eating and lifestyle behaviours (e.g., limiting factors) related to well-balanced diets among Japanese high school students. Additionally, we conducted a simultaneous sex-based multi-population analysis.

As hypothesised, among the female high school students in this study, recollections of *shokuiku* during elementary school years showed a significant positive association with well-balanced dietary habits. This finding is consistent with that of a previous study involving female university students in Japan, which also reported a positive association between recollections of *shokuiku* and well-balanced dietary habits [6]. Furthermore, among female students, the frequency of rice consumption was positively associated with well-balanced dietary habits. Previous research has similarly reported a positive relationship between rice consumption and dietary quality. For instance, data collected in the US over 10 years showed that adults who regularly consumed rice tended to consume less fat and saturated fatty acids and were more likely to consume diets rich in vegetables, dietary fibres, and iron than non-rice consumers [16]. Therefore, the present findings support previous findings. Eating meals with family was the only positive factor common to both sexes and was associated with a well-balanced diet. Although high school students gradually become less dependent on their families as they develop their personalities and expand their spheres of activity, our results underscore the essential role of family influence on eating habits. In addition, the previous finding that late-night snacking had a negative effect on well-balanced dietary habits among female university students living with their families [6], but not among female high school students in the present study, may reflect the role of family support in eating habits.

The 2011 Survey on Time Use and Leisure Activities [17] conducted by the Ministry of Internal Affairs and Communications reported that the average bedtime of Japanese high school students on weekdays was later (23:42) than that of junior high school students (22:55), and that the average sleep duration of high school students on weekdays was correspondingly shorter than that of junior high school students (7 h 09 min vs. 7 h 51 min). Ohida et al. [18] also reported that sleep durations < 6 h are common among high school girls. Previous studies have reported that short sleep duration among adolescents is associated with high consumption of sugary foods and sweetened beverages and lower consumption of fruits and vegetables [9], while eating late at night has been associated with eating low-quality food [10]. Although sleep duration and late-night eating were not directly assessed in this study, the findings suggest that later bedtimes among female high school students may contribute to poorer dietary habits and, consequently, lower adherence to well-balanced dietary habits.

Regarding the association between breakfast frequency and well-balanced diets, we expected to find their association with bedtime; a previous study among subjects aged 40–50 years [19] reported an association between low breakfast frequency and short sleep duration. However, this association was not observed among high school students in the present study. A possible explanation is that the definition of ‘well-balanced dietary habits’ in this study refers to the consumption of balanced meals at least twice a day. Therefore, even if breakfast was consumed infrequently, it would not affect well-balanced diets as long as a complete meal of staple, main, and side dishes was consumed at lunch and dinner. In this study, we found no significant paths from regular breakfast frequency, snacking except for late-night frequency, late dinner, late-night snacking frequency, and regular exercise frequency to well-balanced dietary habits. The absence of a significant association between regular exercise and well-balanced dietary habits may be related to the operational definition of exercise used in this study. Regular exercise was defined as engaging in exercise at least 30 min per session on a regular basis. Consequently, the exclusion of regular exercise lasting <30 min may have influenced the results. Further studies should consider alternative measures of physical activity when examining its relationship with dietary habits.

This study has some limitations. First, the sample was limited to second-year high school students enrolled in a public high school in the Hyogo Prefecture, which may not be representative of all second-year high school students in Japan. Future studies should reduce the influence of random errors due to differences in living environments, such as family, and systematic errors by accounting for factors such as the department, course of study, and regional characteristics when selecting survey targets. Second, because this was a cross-sectional study, data on *shokuiku* during elementary school years were based on participants’ memories of conversations about awareness, attitudes, and practices related to food and nutrition in daily life learned in elementary school. Therefore, we could not assess the actual *shokuiku* during elementary school years and its influence on eating habits in the second year of high school. Third, current dietary and lifestyle behaviours may be influenced by numerous factors that were not assessed in this study, including later-life experiences from elementary school years, family and household environments, and other unobservable factors. Moreover, the present model demonstrated a relatively low coefficient of determination for current well-balanced dietary habits, accounting for only 22% of variance among female high school students. Therefore, the observed associations between current dietary habits and participants’ recollections of *shokuiku* during their elementary school years should be interpreted with caution. Future studies should clarify the *shokuiku* stage and eating and lifestyle behaviours that influence a well-balanced diet and lifestyle through longitudinal studies.

## 5. Conclusions

This study suggests that recollections of *shokuiku* during elementary school years showed a significant positive association with current well-balanced dietary habits among female high school students in the exploratory structural model. Additionally, rice consumption and eating meals with family may be conducive factors to well-balanced dietary habits, unlike a late bedtime. Clarifying the differences in life-course approaches between elementary, junior high, and high school students by sex may be important for the implementation of healthy diets during late adolescence.

## Figures and Tables

**Table 1:** Items potentially associated with well-balanced dietary habits (recollections of *shokuiku* during elementary school years and current eating and lifestyle behaviours) and their interval scales used in the analysis.

Items	Interval Scale	Score (Points)
Well-balanced diets (at least twice daily)		
	6 or 7 d/week	4
	4 or 5 d/week	3
	2 or 3 d/week	2
	≤1 d/week	1
Recollections of *Shokuiku* during elementary school years		
1. Nutritional balance consisting of the ‘three food groups’		
	Agree	4
	Somewhat agree	3
	Do not agree much	2
	Do not agree at all	1
2. Role of major nutrients		
	Agree	4
	Somewhat agree	3
	Do not agree much	2
	Do not agree at all	1
3. Importance of breakfast		
	Agree	4
	Somewhat agree	3
	Do not agree much	2
	Do not agree at all	1
Current eating and lifestyle behaviours		
Regular breakfast frequency		
	6 or 7 d/week	4
	4 or 5 d/week	3
	2 or 3 d/week	2
	≤1 d/week	1
Eating meals with family		
	6 or 7 d/week	4
	4 or 5 d/week	3
	2 or 3 d/week	2
	≤1 d/week	1
Snacking frequency (except for late-night snacking)		
	6 or 7 d/week	4
	4 or 5 d/week	3
	2 or 3 d/week	2
	≤1 d/week	1
Late dinner (2 h before bedtime)		
	6 or 7 d/week	4
	4 or 5 d/week	3
	2 or 3 d/week	2
	≤1 d/week	1
Late-night snacking frequency (from after dinner until bedtime)		
	6 or 7 d/week	4
	4 or 5 d/week	3
	2 or 3 d/week	2
	≤1 d/week	1
Bedtime		
	After 2:00 a.m.	5
	From 1:00 a.m. to 2:00 a.m.	4
	From 0:00 a.m. to 1:00 a.m.	3
	From 11:00 p.m. to 0:00 a.m.	2
	Before 11:00 p.m.	1
Regular exercise frequency (exercise for at least 30 min)		
	6 or 7 d/week	4
	4 or 5 d/week	3
	2 or 3 d/week	2
	≤1 d/week	1

**Table 2:** Participant characteristics and sex-based comparisons (*n* = 254).

Characteristics		Total		Female		Male		*p*
		*n* = 254		*n* = 143		*n* = 111		
		*n*	%	*n*	*n*	%	*n*	
Weight-for-height (%) [12]								
	Tendency toward thinness, ≤−20	4	1.6	2	1.4	2	1.8	1.00 ^†^
	Normal, >−20 and <20	238	93.7	134	93.7	104	93.7	
	Tendency toward obesity, ≥20	12	4.7	7	4.9	5	4.5	
Physical activity level [11]								
	Spending most of the day sitting, Low	47	18.5	33	23.1	14	12.6	<0.001 ^‡^
	Normal	118	46.5	81	56.6	37	33.3	
	Have active exercise habits, High	89	35.0	29	20.3	60	54.1	
Support for healthy eating habits from family and others								
	Fairly much	59	23.2	32	22.4	27	24.3	0.61 ^‡^
	A little	127	50.0	68	47.6	59	53.2	
	Almost none	52	20.5	33	23.1	19	17.1	
	Not at all	16	6.3	10	7.0	6	5.4	
Well-balanced diets								
	6 or 7 d/week	82	32.3	39	27.3	43	38.7	0.008 ^§^
	4 or 5 d/week	89	35.0	50	35.0	39	35.1	
	2 or 3 d/week	62	24.4	37	25.9	25	22.5	
	≤1 d/week	21	8.3	17	11.9	4	3.6	

^†^ Fisher’s exact test; ^‡^ Chi-squared test; ^§^ Welch’s *t*-test, as the data were analysed using interval scales (4 = 6 or 7 d/week; 3 = 4 or 5 d/week; 2 = 2 or 3 d/week; 1 = 1 d/week or less).

**Table 3:** Sex-based comparison of factors potentially associated with well-balanced diets (recollections of *shokuiku* during elementary school years and current eating and lifestyle behaviours; *n* = 254).

Factors Potentially Associated with Well-Balanced Diets		Female		Male		*p*
		*n* = 143		*n* = 111		
		*n*	%	*n*	%	
Recollections of *Shokuiku* during elementary school years						
1. Nutritional balance consisting of the ‘three food groups’						
	Agree	37	25.9	24	21.6	0.16
	Somewhat agree	53	37.1	36	32.4	
	Do not agree much	30	21.0	27	24.3	
	Do not agree at all	23	16.1	24	21.6	
2. Role of major nutrients						
	Agree	36	25.2	27	24.3	0.11
	Somewhat agree	57	39.9	30	27.0	
	Do not agree much	29	20.3	30	27.0	
	Do not agree at all	21	14.7	24	21.6	
3. Importance of breakfast						
	Agree	43	30.1	28	25.2	0.16
	Somewhat agree	62	43.4	46	41.4	
	Do not agree much	24	16.8	20	18.0	
	Do not agree at all	14	9.8	17	15.3	
Current eating and lifestyle behaviours						
Regular breakfast frequency						
	6 or 7 d/week	95	66.4	82	73.9	0.56
	4 or 5 d/week	23	16.1	12	10.8	
	2 or 3 d/week	16	11.2	7	6.3	
	≤1 d/week	9	6.3	10	9.0	
Rice consumption frequency (number per week) ^†^						
	Breakfast	3.5	(2.9)	3.8	(3.8)	0.50
	Lunch	5.7	(2.1)	6.1	(2.7)	0.16
	Dinner	5.7	(2.2)	6.5	(2.9)	0.016
Eating meals with family						
	6 or 7 d/week	63	44.1	53	47.7	0.73
	4 or 5 d/week	46	32.2	27	24.3	
	2 or 3 d/week	24	16.8	19	17.1	
	≤1 d/week	10	7.0	12	10.8	
Snacking frequency (except for late-night snacking)						
	6 or 7 d/week	27	18.9	22	19.8	0.26
	4 or 5 d/week	35	24.5	15	13.5	
	2 or 3 d/week	54	37.8	47	42.3	
	≤1 d/week	27	18.9	27	24.3	
Late dinner (2 h before bedtime)						
	6 or 7 d/week	20	14.0	18	16.2	0.78
	4 or 5 d/week	21	14.7	12	10.8	
	2 or 3 d/week	29	20.3	28	25.2	
	≤1 d/week	73	51.0	53	47.7	
Late-night snacking frequency (from after dinner until bedtime)						
	6 or 7 d/week	20	14.0	19	17.1	0.28
	4 or 5 d/week	17	11.9	13	11.7	
	2 or 3 d/week	38	26.6	36	32.4	
	≤1 d/week	68	47.6	43	38.7	
Bedtime						
	After 2:00 a.m.	4	2.8	5	4.5	0.29
	From 1:00 a.m. to 2:00 a.m.	25	17.5	23	20.7	
	From 0:00 a.m. to 1:00 a.m.	74	51.7	56	50.5	
	From 11:00 p.m. to 0:00 a.m.	30	21.0	21	18.9	
	Before 11:00 p.m.	10	7.0	6	5.4	
Regular exercise frequency (exercise for at least 30 min)						
	6 or 7 d/week	31	21.7	68	61.3	<0.001
	4 or 5 d/week	28	19.6	24	21.6	
	2 or 3 d/week	70	49.0	14	12.6	
	≤1 d/week	14	9.8	5	4.5	

^†^ Values of rice consumption frequency (number per week) are shown as the mean (standard deviation). A Welch’s *t*-test was used, as the data on the factors were analysed as interval-scale variables in the covariance structure analysis. Recollections of *shokuiku* during elementary school years interval scale: 4 = Agree; 3 = Somewhat agree; 2 = Do not agree much; and 1 = Do not agree at all. Bedtime interval scale: 5 = after 2:00 a.m.; 4 = from 1:00 a.m. to 2:00 a.m.; 3 = from 0:00 a.m. to 1:00 a.m.; 2 = from 11:00 p.m. to 0:00 a.m.; and 1 = before 11:00 p.m. Other interval scales: 4 = 6 or 7 d/week; 3 = 4 or 5 d/week; 2 = 2 or 3 d/week; and 1 = ≤ 1 d/week.

## Data Availability

The data used in this study are available upon request from the corresponding author. The data are not publicly available for confidentiality reasons.

## Abbreviations

The following abbreviations are used in this manuscript:

GFI	goodness-of-fit index
AGFI	adjusted goodness-of-fit index
CFI	comparative fit index
RMSEA	root mean square error of approximation
AIC	Akaike’s information criterion
